# Methyl Jasmonate (MeJA) Promotes the Self-Pollen Tube Growth of *Camellia oleifera* by Regulating Lignin Biosynthesis

**DOI:** 10.3390/ijms251910720

**Published:** 2024-10-05

**Authors:** Yihong Chang, Xinmiao Guo, Honggang Xu, Qixiao Wu, Anqi Xie, Zhixuan Zhao, Ruijie Tian, Wenfang Gong, Deyi Yuan

**Affiliations:** Key Laboratory of Cultivation and Protection for Non-Wood Forest Trees of the Ministry of Education, Central South University of Forestry and Technology, Changsha 410004, China

**Keywords:** *Camellia oleifera*, self-incompatibility, methyl jasmonate, lignin, pollen tube

## Abstract

Self-incompatibility (SI) poses a significant reproductive barrier, severely impacting the yield, quality, and economic value of *Camellia oleifera*. In this study, methyl jasmonate (MeJA) was employed as an exogenous stimulus to alleviate SI in *C. oleifera*. The research findings revealed that an exogenous dose of 1000 μmol·L^−1^ MeJA enhanced the germination and tube growth of *C. oleifera* self-pollen and greatly improved ovule penetration (18.75%) and fertilization (15.81%), ultimately increasing fruit setting (18.67%). It was discovered by transcriptome analysis that the key genes (*CAD*, *C4H*) involved in the lignin production process exhibited elevated expression levels in self-pistils treated with MeJA. Further analysis showed that the lignin concentration in the MeJA-treated pistils was 31.70% higher compared with the control group. As verified by pollen germination assays in vitro, lignin in the appropriate concentration range could promote pollen tube growth. Gene expression network analysis indicated that transcription factor *bHLH* may be pivotal in regulating lignin biosynthesis in response to MeJA, which in turn affects pollen tubes. Further transient knockdown of *bHLH* (*Co_33962*) confirmed its important role in *C. oleifera* pollen tube growth. In summary, the application of MeJA resulted in the stimulation of self-pollen tube elongation and enhanced fruit setting in *C. oleifera*, which could be associated with the differential change in genes related to lignin synthesis and the increased lignin content.

## 1. Introductions

*Camellia oleifera* Abel. belongs to the *Camellia* genus and is one of the main tree species utilized for oil extraction in China [1]. *Camellia* oil has a high dietary property and medicinal value with a substantial amount of oleic acid, monounsaturated fatty acid, green tea polyphenols, and tea saponin [2,3], which has a significant effect on preventing such as hypertension, hyperlipidemia, hepatocellular carcinoma, and other diseases [4,5]. It has been listed by the Food and Agriculture Organization of the United Nations as one of the popularized healthy edible oils [6]. Its by-products have extensive applications in the chemical fiber, daily chemical, and lithium battery industries, including fruit shells, seed cakes, and tea meals [7,8,9]. With growing recognition of its nutritional value, the social demand for *Camellia* oil has increased substantially. However, the *C. oleifera* industry often faces challenges such as low fruit set and yield, and one of the important reasons for this is self-incompatibility (SI) [10,11]. Hence, the disruption of the SI mechanism in *C. oleifera* stands out as a crucial area of focus for enhancing the quality and production within the industry.

SI is an intraspecific evolutionary strategy, which allows pistils to identify and discard self-pollen/pollen tubes, thereby avoiding inbreeding depression and encouraging cross-pollination [12,13,14]. *C. oleifera* SI is characterized by pollen and styles that can normally recognize, hydrate, and elongate, but most pollen tubes are hindered at the bottom of the style and accompanied by the enlargement and bending of the pollen tube apex [10,15]. In recent times, with the vigorous advancement of genetic engineering, modern biotechnology such as breeding by gene silencing and genome editing has been used to break down SI for crop improvement. Ye et al. [16] employed CRISPR/Cas9 technology to target and modify *S-RNase* genes, resulting in the disruption of SI mechanisms and successful transitions from SI to self-compatibility (SC) in potato plants. Moreover, genetic transformation and RNAi were also applied to overcome SI for crop improvement [17,18,19]. Unfortunately, in *C*. *oleifera*, *S*-determinants have remained a mystery for years and a transgenic system is lacking. Therefore, it is difficult to manipulate the target SI factors directly using biotechnology to break SI like in other self-incompatible crops. In addition, methods such as temperature stress, mentor pollination, and bud pollination have also been used to increase the production of SI plants [20,21]. However, these methods cannot be used on a large scale in production because of their time-consuming and labor-intensive nature. Hence, exploring more effective ways to break SI has become one of the important issues. Recently, plant exogenous inducers, such as Tanase (TA), 1-Naphthaleneacetic acid (NAA), Indole acetic acid (IAA), I-aminocyclopropane carboxylic acid (ACC), and Gibberellic acid (GA), have been confirmed to affect pollen tube growth and effectively improve crop yield [22,23,24,25,26,27]. Our previous study found that exogenous methyl jasmonate (MeJA) could effectively promote pollen tube growth in vitro [28].

MeJA belongs to the jasmonic acid (JA) class of compounds, which is ubiquitous in different plant tissues. As a signaling molecule, it participates in various physiological processes in plants [29,30,31]. Previous studies indicated that the effect of the external application of MeJA induced the accumulation of terpenoids, especially phenylethyl alcohol, linalool, and its oxides [32]. The exogenous spraying of MeJA promoted the growth of seeds and fruits and regulated the biosynthesis of unsaturated fatty acids and the total oil content in *C. oleifera* seeds [33]. Recent findings found that spraying MeJA improved the root growth of pigeon pea and promoted phenol and flavonoid synthesis [34]. Moreover, MeJA could play multiple roles in plants’ pollen tube growth. Muradoğlu, et al. [35] reported that JA and MeJA treatment (0.05–1 mM) could inhibit the elongation of apricot pollen tubes and activate the expression of genes associated with the JA signaling pathway. Conversely, other scholars showed that 0.05 mM MeJA could stimulate pollen sprouting and tube extension in *Pinus nigra* [36]. Liu et al. [37] found that a high concentration of MeJA inhibited pollen tube growth of *C. oleifera* in vitro. These research studies indicate that the impact of MeJA on pollen growth is contingent upon both the dosage and the specific species involved.

Despite the availability of various studies exploring the impact of MeJA on pollen tube growth in vitro, few studies have been conducted on the effects of MeJA on the growth behavior of self-pollen tubes and fruit yield in *C. oleifera,* with the underlying molecular mechanisms remaining elusive. In order to explore an effective way to overcome SI, we examined the effects of applying exogenous MeJA on the self-pollen tubes in *C. oleifera*. The effects of MeJA treatment on the growth behavior of the self-pollen tubes were analyzed and discussed through anatomical and cytological methods. Concurrently, the expression of associated genes within the phenylpropanoid pathway and the synthesis of metabolites were also analyzed and explored by combining transcriptome sequencing and biochemical determination analysis. This work provides a novel perspective on the potential application of MeJA in other SI plants.

## 2. Results

### 2.1. Appropriate Concentrations of MeJA Promoted Pollen Germination and Pollen Tube Elongation

As shown in Table 1, the germination rate of MeJA-treated pollen grains was significantly higher than that of the control. Specifically, it increased most significantly at 50 μmol·L^−1^, followed by 5 μmol·L^−1^, with an improvement of 10.36% and 7.88% compared with the control, respectively. Similarly, the length of the pollen tube in 2 h increased by 42.76% at 50 μmol·L^−1^ and by 28.10% at 5 μmol·L^−1^ compared with the control. When pollen tubes were grown for 48 h, 5 μmol·L^−1^ of MeJA had a weak positive effect on pollen tube length, but 50 μmol·L^−1^ of the MeJA treatment significantly reduced the pollen tube length by 50.97% compared with the control. These results indicated that the appropriate dose of MeJA could positively affect both pollen sprouting and pollen tube extension. A high concentration of MeJA actually inhibited the growth of pollen tubes, and 5 μmol·L^−1^ appeared to be the ideal working concentration to improve the pollen germination rate and pollen tube growth in vitro.

### 2.2. MeJA Improved the Fruit Setting Percentage in Self-Pollinated C. oleifera

**Figure 1:**
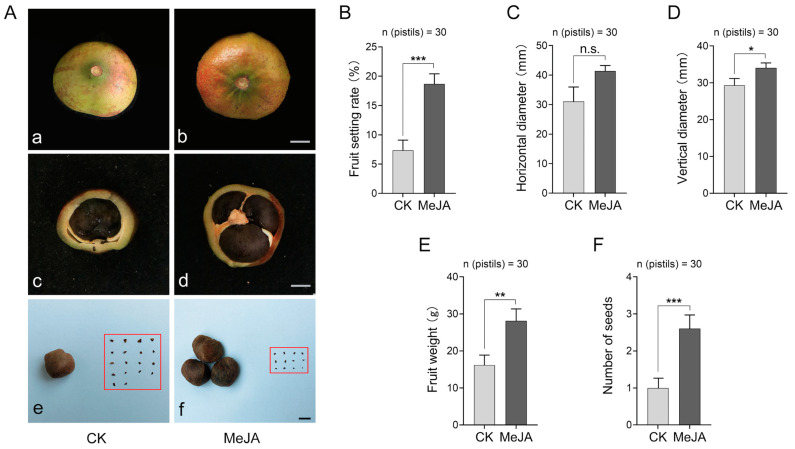
Effects of the external application MeJA on the fruit of *C. oleifera*. (**A**) Phenotypic contrast of ripe fruits under the 1000 μmol·L^−1^ MeJA treatment. The red block shows abortive seeds. Bars = 5 cm (**a**–**d**). Bars = 1 cm (**e**,**f**). (**B**–**F**) The fruit phenotype statistics. “n” indicates the statistical number of fruits. * *p* < 0.05, ** *p* < 0.01, *** *p* < 0.001 (*t*-test). “n.s.” indicates no significance. The error bars display the standard error of the sample. MeJA represents the treatment with 1000 μmol·L^−1^ methyl jasmonate solution for *C. oleifera* self-pollinated pistils, and CK signifies the treatment with a solution without methyl jasmonate.

The fruit setting rate is the most intuitive indicator reflecting the strength of compatibility. In pre-experiments, we sprayed different concentrations of MeJA to explore the effect on the fruit setting percentage in self-pollinated *C. oleifera* according to Song et al. [33] (Table S1), which showed that 1000 μmol·L^−1^ had the highest fruit setting rate. Further counting of the fruits revealed that the 1000 μmol·L^−1^ MeJA treatment resulted in larger fruits and an increased number of full seeds (Figure 1A). In comparison with the control, the MeJA treatment remarkably increased the fruit setting rate by 11.34% (Figure 1B). The horizontal and vertical diameter of the fruit increased by about 24.92% and 13.87% (Figure 1C,D), respectively, while the fruit weight increased accordingly (42.42%) (Figure 1E). Importantly, the number of seeds per fruit exhibited a significant two-fold increase compared with the control (Figure 1F). Together, the MeJA treatment could improve fruit production in self-pollinated *C. oleifera.*

### 2.3. MeJA Increased the Rate of Ovule Penetration and Fertilization

**Figure 2:**
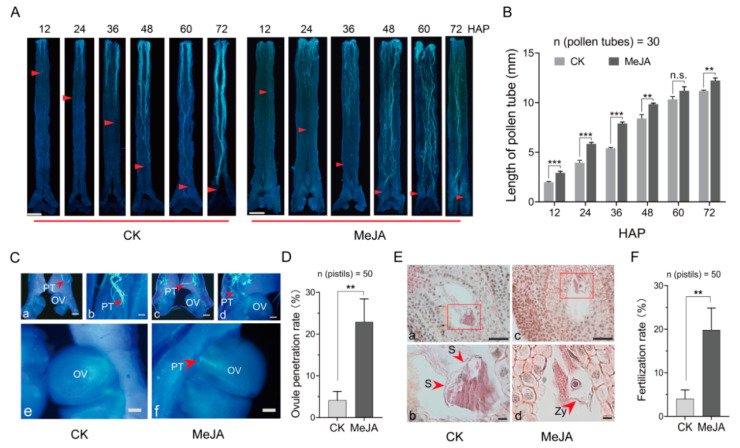
Effects of MeJA on the growth of *C. oleifera* self-pollen tubes in vivo. (**A**) Growth patterns of self-pollen tubes in both the control and MeJA treatments. The red arrow indicates the position of the pollen tube. Bar = 1000 μm. (**B**) Measurement of pollen tube length at different time points. “n” represents the statistical number of pollen tubes. (**C**) The ovule penetration of the self-pollen tube. OV: ovule; PT: pollen tube. The red arrow indicates the pollen tube. Bar = 100 μm (**a**–**d**). Bar = 300 μm (**e**,**f**). (**E**) The fertilization status of self-pollination. (**b**) and (**d**) are the enlarged images shown in the red boxes in (**a**) and (**c**) respectively. (**b**,**d**) are partially enlarged views of (**a**,**c**), respectively. S: synergid; Zy: zygote. Bar = 50 μm. (**D**,**F**) Statistics on the penetration of the ovule and double fertilization. “n” represents the statistical number of pistils. ** *p* < 0.01, *** *p* < 0.001 (*t*-test). “n.s.” indicates no significance. The error bars display the standard error of the sample. MeJA represents the treatment with 1000 μmol·L^−1^ methyl jasmonate solution for *C. oleifera* self-pollinated pistils, and CK signifies treatment with a solution without methyl jasmonate.

To further test whether MeJA affects pollen tube elongation in vivo, we observed the pollen tube length in the style treated with 1000 μmol·L^−1^ MeJA. The results showed that the pollen tube growth with MeJA treatment was observably faster than that with the control (Figure 2B). After pollination, the progress of the pollen tube in the control group slowed or even stopped at the base of the style around 48 h, accompanied by an incompatibility phenotype such as curly and wavy. However, after MeJA treatment, the pollen tubes continued to elongate and enter the ovule, with alleviation of the incompatibility phenotypes (Figure 2A). We further explored the pollen tube growth in the ovary after MeJA treatment. The fluorescence microscope showed that the ovule penetration rate was significantly increased by 18.75% following MeJA treatment compared with the control group (Figure 2C,D). The fertilization rate increased by up to 19.85% after MeJA treatment (Figure 2E,F). These data demonstrated that MeJA treatment improves the performance of self-pollen tubes in vivo compared with the control, indicating that MeJA treatment has a positive effect on breaking SI of *C. oleifera*.

### 2.4. Differential Expression of Pistil Genes in C. oleifera Treated with MeJA

**Figure 3:**
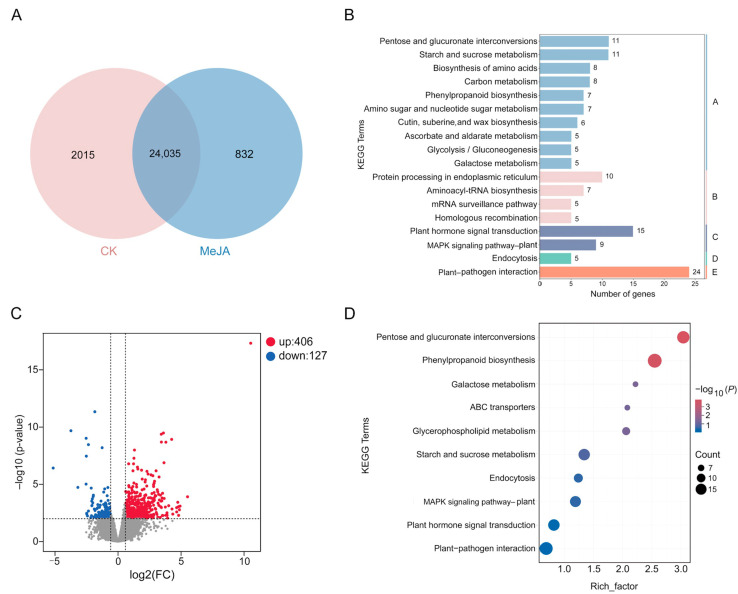
Transcriptomic analysis of *C. oleifera* self-pistils under MeJA treatment. (**A**) Venn diagram of the control group and the 1000 μmol·L^−1^ MeJA-treated *C. oleifera* pistil samples. The central overlap region indicates the unigene numbers shared between the two groups. (**B**) KEGG classification of 832 genes unique to the MeJA treatment group (based on the number of genes ≥ 5). A: metabolism; B: genetic information processing; C: environmental information processing; D: cellular processes; E: organismal systems. (**C**) Volcano plot of DEGs between the two groups. The gray dots represent non-DEGs, and the gray dashed lines are used to draw the distinction between DEGs and non-DEGs. (**D**) Top 10 terms from the KEGG enrichment analysis of DEGs. The ‘rich factor’ indicates the ratio of DEGs enriched in a specific KEGG term to the total number of annotated unigenes within that KEGG term. A larger rich factor signifies a higher degree of enrichment. MeJA represents the treatment with 1000 μmol·L^−1^ methyl jasmonate solution for *C. oleifera* self-pollinated pistils, and CK signifies treatment with a solution without methyl jasmonate.

Transcriptome analysis was carried out using the pistils treated with and without 1000 μmol·L^−1^ MeJA. We obtained 26,882 unigenes, among which 832 were unique to those with MeJA treatment (Figure 3A). Based on the KEGG classification, 832 genes unique to the MeJA treatment group were categorized into five types (Figure 3B) as follows: genetic information processing (66), organismal systems (24), metabolism (185), cellular processes (12), and environmental information processing (29). Among them, the number of branching routes in the ‘metabolism’ category was the largest, and the top five in this pathway were ‘pentose and glucuronate interconversions’, ‘starch and sucrose metabolism’, ‘biosynthesis of amino acids’, ‘carbon metabolism’, and ‘phenylpropanoid biosynthesis’, respectively. There were 2015 genes expressed only in the control group, which were insensitive to MeJA treatment and related to secondary metabolism, RNA transport, etc. (Figure S1). Compared with the control group, 533 differentially expressed genes (DEGs) were discovered after MeJA treatment, of which 406 genes were up-regulated and 127 genes were down-regulated (Figure 3C, Table S3). The KEGG entries in the top 10 of their enrichment are shown in Figure 3D. Based on the results, ‘pentose and glucuronate interconversions (ko00040)’ and ‘phenylpropanoid biosynthesis (ko00940)’ were significantly enriched. Moreover, a significant proportion of DEGs was enriched in the phenylpropanoid biosynthesis pathway, indicating a potential pivotal role of this pathway in response to MeJA treatment.

### 2.5. DEGs in Phenylpropanoid Biosynthesis

**Figure 4:**
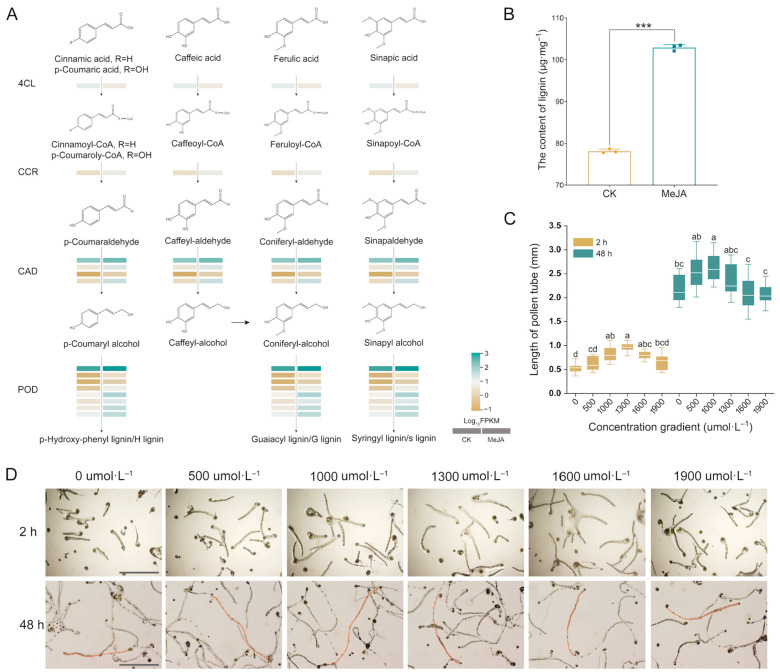
Analysis of the phenylpropane pathway and lignin metabolism. (**A**) Differential expression of phenylpropanoid biosynthesis pathway enzyme genes under MeJA treatment. Each row corresponds to a DEG, and colors from yellow to green indicate that the levels of gene expression are low to high. POD: peroxidase; CAD: cinnamyl alcohol dehydrogenase; CCR: cinnamoyl-CoA reductase; 4CL: 4-coumarate-CoA ligase. The black arrow indicates the metabolic direction, pointing to the next metabolite. (**B**) Lignin content in the pistil of *C. oleifolia*. *** *p* < 0.001 (*t*-test). (**C**) The length measurement of *C. oleifera* pollen tube after different concentrations of lignin treatment in vitro for 2 h and 48 h. Different letters represent significant differences by one-way ANOVA with Tukey’s post hoc test (*p* < 0.05). The error bars display the sample standard error. n (pollen tubes) = 30. (**D**) Comparison of *C. oleifera* pollen tube length after various lignin concentration treatments. The red free line represents the pollen tube. Bars = 1 mm. MeJA represents the treatment with 1000 μmol·L^−1^ methyl jasmonate solution for *C. oleifera* self-pollinated pistils, and CK signifies treatment with a solution without methyl jasmonate.

Based on the KEGG analysis results, we further integrated the phenylpropanoid biosynthesis pathway. The structural genes related to the biosynthesis and metabolism of lignin, such as *4-coumarate-CoA ligase* (*4CL*), *cinnamoyl-CoA reductase* (*CCR*), *cinnamyl alcohol dehydrogenase* (*CAD*), and *peroxidase* (*POD*), exhibited differential expression (Figure 4A). The expression levels of one *CCR*, four *CADs*, and eight *PODs* exhibited a significant increase, while one *4CL* was downregulated after MeJA treatment. In addition, the lignin content exhibited a notable increase following MeJA treatment in comparison with the control group, aligning with the expression trend in structural genes (Figure 4B). We also performed pollen tube germination assays in vitro using different concentrations of lignin. As shown in Figure 4C,D, exogenous lignin could considerably promote the extension of pollen tubes in a dose-dependent manner, and the optimal concentration ranged from 1000 μmol·L^−1^ to 1300 μmol·L^−1^.

### 2.6. Regulatory Network of Key Genes with Transcription Factors

**Figure 5:**
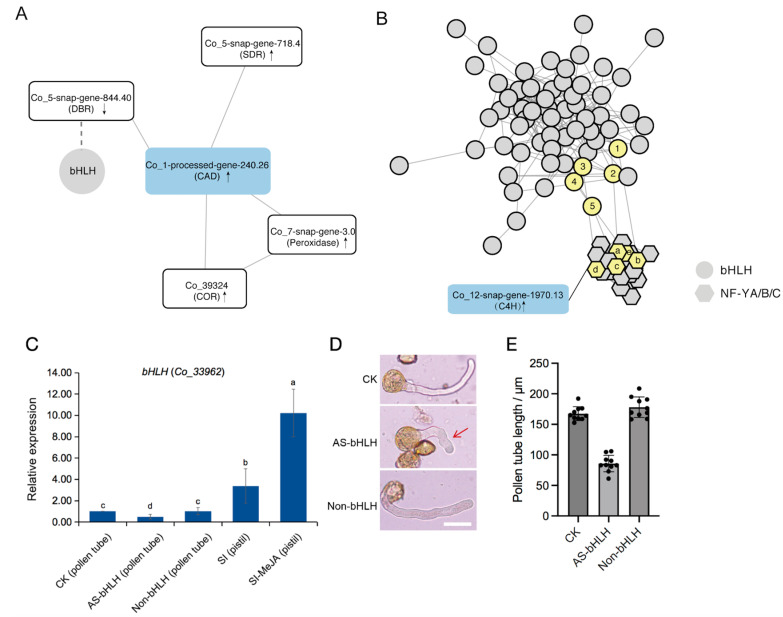
Regulatory network and functional analysis of hub genes. (**A**) Analysis of the correlation between the gene of lignin synthesis and all DEGs except TFs. (**B**) The regulatory network between the gene of lignin synthesis and all the predicted TFs. Black arrows represent up- and down-regulated. (**C**) Expression of the *bHLH* (*Co_33962*) gene in pollen tubes and self-pollinated pistils. AS-bHLH indicates that AS-ODN treatment significantly reduced the expression of the *bHLH* gene in *C. oleifera* pollen tubes relative to the control (CK and non-bHLH) (**D**) Phenotypes of AS-ODN-treated and control pollen tubes. Bar = 50 μm. The red arrow indicates that knocking down *bHLH* (*Co_33962*) expression reduces the length of pollen tubes. (**E**) Length of AS-ODN-treated and control pollen tubes. Different lowercase letters indicate significant differences between groups (*p* < 0.05, ANOVA). Note, 1–5: *bHLH126* (*Co_11-snap-gene-234.21*), *bHLH74* (*Co_19887*), *bHLH* (*Co_33962*), *UNE10* (*Co_1-snap-gene-1816.38*), *bHLH35* (*Co_4-snap-gene-1101.68*); a–e: *NFYA2* (*Co_30119*), *NFYB4 (Co_1-processed-gene-839.0)*, *NF* (*Co_30184*), *NFYB3* (*Co_2-processed-gene-1850.1*), *NFYC3* (*Co_12625*). AS: Antisense Oligodeoxynucleotide; Non: nonsense oligodeoxynucleotide. SI: self-incompatibility.

To better understand the regulatory mechanisms of lignin metabolism-related genes affecting pollen tube growth, the STRING method was used to explore the transcriptional regulatory network of selected DEGs. With *CAD* (*Co_1-processed-gene-240.26*) as the hub gene of the network, five DEGs that contributed to the lignin synthesis had the strongest connectivity (Figure 5A), and the qRT-PCR assay verified the expression changes in these genes in response to MeJA treatment (Figure S2), indicating their effectiveness in controlling changes in lignin content and pollen tube growth. Similarly, different members of the transcription factor family *NF-Ys* were found to interact with *C4H* (*Co_12-snap-gene-1970.13*) (Figure 5B), which were significantly up-regulated in MeJA-treated *C. oleifera* pistil (Figure S2). After further exploring the association between the *CAD*-centered network and the *C4H*-centered network, we found that different members of the *bHLH* family can interact with genes in the two networks (Figure 5A,B). Among them, qRT-PCR showed that *bHLH* (*Co_33962*) was also significantly up-regulated in self-pistils in response to MeJA treatment (Figure 5C). To validate the function of the hub gene *bHLH* (*Co_33962*) for *C. oleifera* pollen tubes, an additional Antisense Oligodeoxynucleotide (AS-ODN) experiment was conducted. It revealed that transient knockdown of *bHLH* (*Co_33962*) led to a significant reduction in pollen tube length (Figure 5C–E), verifying its pivotal role in facilitating pollen tube elongation. This finding not only reinforced our previous transcriptome and qPCR data but also provided a more direct and pertinent functional validation in *C. oleifera*. The above results suggested that transcription factors, including *bHLH* (*Co_33962*), likely served as the bridge, modulating the expression of key structural genes in response to MeJA stimuli and ultimately influencing pollen tube growth in *C. oleifera*.

## 3. Discussion

In angiosperms, SI serves as a prevalent mechanism to prevent self-fertilization, enabling plants to identify and discard self-pollen, thereby inhibiting pollen tube elongation post-germination and promoting genetic variability. *C. oleifera* exhibits self-incompatibility traits, leading to the cessation of pollen tube growth at the style following self-pollination for 48 h. This is accompanied by observable phenotypes such as swelling, wavy, and other signs of incompatibility at the pollen tube tip. The development of pollen tubes is a prerequisite that influences fertilization and seed set. Therefore, it is important to explore effective methods to overcome SI and improve the yield of *C. oleifera*.

### 3.1. Effect of MeJA on the Development of Pollen Tubes

MeJA is a multifunctional growth regulator extensively present in plants. Its exogenous application has a crucial effect on regulating pollen tube growth, anther dehiscence, seed germination, and others. In this study, the exogenous application of MeJA was performed to investigate the development of *C. oleiferae* pollen tubes. The results of the in vitro experiments showed that the effects of MeJA treatment on pollen germination and tube growth exhibited a dose-dependent pattern. The different concentrations of MeJA significantly promoted the sprouting and tube elongation of pollen after 2 h, and the most stimulating effect was observed at 5 μmol·L^−1^. However, 50 μmol·L^−1^ markedly inhibited pollen tube elongation after 48 h of incubation. Similar results were observed in apricots. According to a study by Muradoğlu, Yıldız, and Balta [35], pollen sprouting and tube elongation in apricots were highly inhibited following treatment with MeJA at concentrations of 0.5 mM and 1 mM, while they slightly increased at 0.1 mM and 0.25 mM concentrations. Also, exogenously supplied MeJA at 0.05 and 0.25 mM concentrations had a promoting effect on pollen germination in *Pinus nigra*, while no germination was noted following treatment with 2.5 mM MeJA [36]. Thus, appropriate doses of MeJA could have beneficial effects on pollen sprouting and tube elongation, while excessive levels could inhibit the development of the tubes. Field spraying is applied directly to trees, allowing for intuitive observations of the effect of MeJA, thereby avoiding artificial differences triggered by in vitro culture. Studies using MeJA spraying in the field have been reported for seed development, fruit quality, and substance synthesis. For example, foliar spraying of MeJA improved the productivity and standard of fragrant rice by inducing the biosynthesis of 2-acetyl-1-pyrroline (2-AP) [38]. Researchers reported that exogenously applied 0.5 mM MeJA resulted in a notable increase in the dimensions of *C. oleifera* fruits and seeds [33]. However, the effects of MeJA application on the pollen tube development in vivo and fruit setting of *C. oleifera* were not reported. Hence, the current study demonstrated that a 1000 μmol·L^−1^ MeJA treatment observably promoted the internal growth of the pollen tube and ovule penetration and improved the fertilization rate, thus efficiently alleviating the SI of *C. oleifera*. These results provided valuable insights into the potential application of MeJA in other SI plants.

### 3.2. Effect of MeJA on Lignin Biosynthesis

MeJA not only participates in the physiological processes of plants as a regulator but also acts as an activator to induce the production of secondary metabolites in plants. The exogenous application of MeJA induces plants to synthesize a variety of compounds such as terpenes, ketones, phenols, alcohols, etc. Feng, et al. [39] found that exogenous MeJA improved the flavor of water dropwort by promoting the production of terpenoids. After spraying MeJA, the structural genes of flavonoid synthesis in *C. oleifera* were significantly regulated, thereby promoting the synthesis and accumulation of multiple flavonoids in seeds [40]. Moreover, the exogenous application of MeJA can also promote lignin biosynthesis and deposition in the pathway by activating the activity of related enzyme genes in the phenylpropanoid pathway. Previous studies showed that exogenous MeJA treatment strengthened the PAL, C4H, 4CL, and CAD enzyme activity in kiwifruit and increased the lignin content [41]. Comparable findings have been documented in *Camellia sinensis* and peaches [42,43]. Our studies aligned with these reports. MeJA treatment activated the signaling of the phenylpropanoid pathway and structural genes related to synthesis by catalyzing various substrates in the pathway. We speculated that the deposition of lignin on the pollen tube cell wall increased the mechanical support strength of the pollen tube, consequently facilitating the elongation of self-pollen tubes. Studies on the promotion of lignin biosynthesis by MeJA have reported plant disease resistance, fruit preservation after harvest, and others. However, there is no study on the effect of MeJA in alleviating the SI of *C. oleifera* by accumulating lignin, and our results offer valuable perspectives for further investigations.

### 3.3. The Regulatory Mechanism of Lignin Biosynthesis

**Figure 6:**
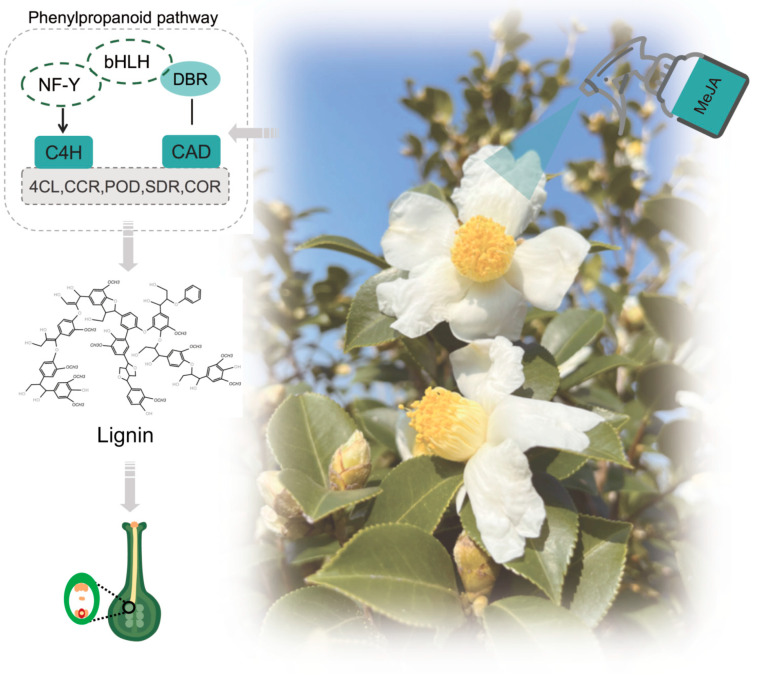
Model of the regulation of C. oleifera self-pollen tubes by MeJA. bHLH, basic Helix–Loop–Helix; NF-Y, Nuclear factor Y; DBR, 2-alkenal reductase; C4H, Cinnamate 4-hydroxylase; CAD, cinnamyl alcohol dehydrogenase; 4CL, 4-coumarate-CoA ligase; CCR, cinnamoyl-CoA reductase; POD, peroxidase; SDR, short-chain dehydrogenase/reductase; COR, codeinone reductase.

Lignin is a component found in the plant cell wall, which helps to enhance the mechanical strength of plants [44]. The phenylpropanoid metabolism pathway and the specific pathway responsible for lignin synthesis are recognized as the primary routes involved in the production of lignin monomers [45,46]. Studies have shown that a variety of key enzymes are involved in both pathways, including PAL, CAD, 4CL, C4H, CCR, and others [45,46]. Nine *CAD* genes were isolated from the *Arabidopsis* genome, and three of them (*AtCAD1*, *AtCAD4*, and *AtCAD5*) were identified as the main enzymes in the lignin biosynthesis pathway [47]. Many plants respond to various adverse conditions by synthesizing lignin and increasing the expression of the *CAD* gene at an early stage [48]. Zhong et al. [49] suggested that the *PpCAD* promoter of elephant grass is involved in lignin deposition via responding to molecular signaling from MeJA. C4H has also been shown to be involved in lignin biosynthesis and to be able to respond to hormonal treatments [50,51]. Regarding the transcriptional regulation of lignin metabolism core genes, it was shown that *AabHLH113* was able to alter artemisinin biosynthesis in *Artemisia annua* by regulating *DBR2* in response to MeJA signaling [52]. *bHLH* was shown to have a broad interaction with *NF-Ys* as well [53,54]. In this research, the level of hub genes *C4H*, *CAD*, *NY-Fs*, and *bHLH*, except for *DBR* expression, showed a positive regulatory relationship with lignin accumulation trends after MeJA treatment. We also found transcriptional regulatory relationships directly between *bHLH*, *NF-Ys,* and *C4H*. To sum up, it can be inferred, that *bHLH* might accelerate the rate of lignin biosynthesis by regulating *DBR/CAD* and *NF-Ys/C4H* molecular modules (Figure 6). In combination with known structure genes, it is inferred that lignin biosynthesis was not caused by a single but by a series of transcription factors and key structure genes, which together form a regulatory network coordinated and inhibited with each other. Further study is required to identify a deeper mechanism of action.

## 4. Materials and Methods

### 4.1. Plant Material and MeJA Treatment (Pollen Tube Growth In Vivo)

The *C. oleifera* material we used was the national authorized variety ‘Huashuo’ (‘HS’). It was planted in the experimental base located at Liuyang, Hunan, China (113°42′ E, 28°38′ N). Healthy 8-year-old grafted trees were selected for this study. Experiments were carried out when the plants were at the flowering stage between November and December 2022. One liter of 1000 μmol·L^−1^ MeJA working solution with 0.2% of Tween 20 was sprayed on the plant materials on sunny mornings (9:00~11:00), while the control group was treated with water with 0.2% of Tween 20. Each treatment included three plants. After the solution was fully absorbed, artificial self-pollination was performed. On the branches growing vigorously in all directions in the middle of the crown, the buds that were about to open were randomly selected for artificial pollination and marked. One additional spray was applied 3 and 7 days after pollination. Each biological replicate contained 150 pistils and three replicates were designed, of which 50 were counted to determine the fruiting rate after ripening and the rest were used in morphological, physiological, and molecular experiments. The materials for physiological and molecular experiments were stored at −80 °C.

### 4.2. Pollen Tube Growth In Vitro

Pollen grains were collected from ‘HS’ fresh anthers and dried at 26 °C for 12 h [55]. The base medium components were agarose (10 g·L^−1^), sucrose (100 g·L^−1^), H_3_BO_3_ (0.1 g·L^−1^), and CaCl_2_ (0.05 g·L^−1^). Pollen grains in the culture medium supplemented with 0.5 μmol·L^−1^, 5 μmol·L^−1^, and 50 μmol·L^−1^ MeJA were then incubated at 25 ± 2 °C in the dark, and the base medium was used as control. Germination was defined when tube lengths were greater than the diameter of the pollen grains and counted after 2 h. The length of pollen tubes was measured at 2 h and 48 h. The germination of pollen tubes was observed using an Olympus BX-53 light microscope (Olympus, Tokyo, Japan). Each experimental group was composed of three biological replicates.

### 4.3. Aniline Blue Staining of Pollen Tubes

The pistils were gathered and soaked in Carnoy’s solution for 12 h. Subsequently, vacuum extraction was performed for 20 min and stored in 70% alcohol at 4 °C. Following Gao et al. [56], the pistils were hydrated with a series of ethanol with concentrations of 70%, 50%, and 30%, and dissected to separate each style and its corresponding ovary. The tissues were then softened with 8 M NaOH and NaClO for a duration of 2 h and soaked in a 0.2% (*w*/*v*) solution of aniline blue for a duration of 24 h. The growth pattern of the pollen tubes was observed utilizing a fluorescence microscope (Olympus BX-53, Tokyo, Japan).

### 4.4. Fertilization Observation

The pistils were harvested at 150 HAP and fixed using the above method. Fertilization was observed using the paraffin section [57], and the specific steps were as follows: firstly, the villi on the ovary wall and the style were removed from the fixed pistils. Secondly, the materials underwent dehydration using a series of ethanol solutions, cleared in xylene, steeped in wax, then paraffin-embedded materials, and cut into 10 μm slices. Finally, the sample slides were dyed for 15–20 min using 1% ammonium ferric sulfate, followed by rinsing with tap water for 1.5–2 h and roughly 10 h of the dyeing process with hematoxylin. After rinsing for 30 min in tap water, the sections were stained with 0.5% ammonium ferric sulfate for 5–10 min. The slides were dehydrated with an alcohol series and stained with 0.2% eosin. The samples were sealed with neutral gum and observed via an Olympus BX-53 light microscope (Olympus, Tokyo, Japan).

### 4.5. Determination of the Lignin Content

Approximately 0.1 g of the pulverized and desiccated sample was dissolved in 1.5 mL of 70% ethanol solution, mixed thoroughly, and then the supernatant was discarded. The residue was then washed sequentially with 1.5 mL miscible liquids of chloroform/methanol (1:1 *v*/*v*) and acetone and dried under vacuum. Then, 1.5 mL of 0.1 M sodium acetate buffer (pH 5.0) was added and placed for 20 min at 80 °C. Subsequently, 0.5 mL 0.01% sodium azide (NaN_3_), pullulanase, and α-amylase were added and incubated for 24 h at 37 °C. The suspension was heated for 30 min at 100 °C and 1.5 mL of acetone was added after centrifugation (Avanti JXN-26, Shanghai, China); then, the acetone was vacuum-dried. This was followed by adding 300 μL of acetyl bromide solution and heating for 2 h at 37 °C. Then, 480 μL of 2 M sodium hydroxide and 210 μL of 0.5 M hydroxylamine hydrochloride were mixed, and acetic acid was added to 2.0 mL. The absorbance of the supernatant was quantified using Elisa (Multiskan GO, Shanghai, China) at a wavelength of 280 nm in order to determine the lignin content.

$$\mathrm{Formula}:\;\mathrm{Total}\;\mathrm{lignin}\;\mathrm{content}\;(\mu g\;\cdot {\mathrm{mg}}^{-1})=\frac{ABS}{Coeff\times 0.539\;\mathrm{cm}}\times \frac{2\;\mathrm{mL}}{Weight}\times 100\%\times 10$$

where *ABS* is the absorbance, *Coeff* is the coefficient of absorbance, *Weight* is in Mass/mg, and 0.539 cm is the path length [58].

### 4.6. Transcriptome Analysis

#### 4.6.1. RNA Preparation and Transcriptome Sequencing

Total RNA was extracted separately from *C. oleifera* pistils treated with 1000 μmol·L^−1^ MeJA (MeJA, three biological replicates) and water (CK, three biological replicates) 48 h after pollination. Sequencing libraries were generated using the Hieff NGS Ultima Dual-mode mRNA Library Prep Kit for Illumina (Yeasen Biotechnology (Shanghai, China) Co., Ltd.), and index codes were added to attribute sequences to each sample. The libraries were sequenced on an Illumina NovaSeq platform to generate 150 bp paired-end reads. After quality control, the Hisat2 tool software was used to map the data with the reference genome [59]. The gene expression value was quantified as FPKM (Fragments Per Kilobase of transcript per Million fragments mapped). FPKM = Fragment/(total reads/1,000,000)/(transcript/1000). Gene function was annotated based on the following databases: Nr (NCBI non-redundant protein sequences); Pfam (Protein family); KOG/COG (Clusters of Orthologous Groups of proteins); Swiss-Prot (A manually annotated and reviewed protein sequence database); KO (KEGG Ortholog database); and GO (Gene Ontology). The possible transcription factors (TFs) in expressed genes were predicted by the plantTFDB database (https://planttfdb.gao-lab.org (accessed on 4 January 2023)) using hmmsearch software (3.3.2).

#### 4.6.2. Screening and Analysis of the Differentially Expressed Genes

Differentially expressed genes (DEGs) were analyzed by DESeq between the control and MeJA-treated groups. DESeq2 provides statistical routines for determining differential expression in digital gene expression data using a model based on the negative binomial distribution. The resulting *p*-values were adjusted using Benjamini and Hochberg’s approach for controlling the false discovery rate. Genes with an adjusted Fold Change (FC) ≥ 1.5 and *p*-value < 0.01 were assigned as differentially expressed (Table S3). We used the KOBAS database (https://bioinformaticshome.com/tools/rna-seq/descriptions/KOBAS.html (accessed on 4 January 2023)) and clusterProfiler software (4.4.4) to test the statistical enrichment of DEGs in KEGG pathways. The sequences of the selected gene sets were blast (blastx) to the genome of a related species (the protein–protein interaction of which exists in the STRING database at http://string-db.org/ (accessed on 4 January 2023)) to obtain the predicted interactions among these genes [60]. Then, the interactions among these genes were visualized in Cytoscape3.8.2.

### 4.7. Gene Function Verification with the Antisense Oligodeoxynucleotide (AS-ODN) Method

The experimental method followed that described by Liao et al. [61] and Mizuta et al. [62] with appropriate adjustments. The mRNA sequence of *bHLH* (*Co_33962*) was used to predict the secondary structure by RNAfold WebServer (http://rna.tbi.univie.ac.at/cgi-bin/RNAWebSuite/RNAfold.cgi (accessed on 19 August 2024)), and an AS-ODN sequence was designed. The interference efficiency of candidate AS-ODN on *bHLH* expression was evaluated by Soligo (https://sfold.wadsworth.org/cgi-bin/soligo.pl). The candidate AS-bHLH (C*C*G*GTCATGAATGTAAT*C*A*G) and nonsense oligodeoxynucleotide (Non-ODN) (C*C*G*TGACCTGCACGA*C*G*C) sequences were synthesized by Beijing Tsingke Biotech Co., Ltd. (Beijing, China), and the 5’ and 3’ ends of each sequence were thiomodified and purified by HPLC.

The synthesized AS-ODN and Non-ODN sequences were dissolved in a pollen tube culture solution (10% sucrose, 0.01% boric acid, 0.005% CaCl2, and pure water [*w*/*v*]) separately. The ODN solution was transferred into the pre-cultured pollen solution until the final concentration of ODNs was 50 μM. The pollen tube phenotype was observed after continued culture for 3 h. Total RNA was extracted from the pollen tubes after 3 h of ODN treatment to detect the expression of *bHLH*. There were three biological replicates per treatment.

### 4.8. qRT-PCR Analysis

Six representative genes associated with lignin synthesis and three TFs were chosen for qRT-PCR analysis. Pistil samples were from MeJA-treated *C. oleifera* self-pollinated pistils and controls, while pollen tube samples were from AS-ODN-treated and Non-ODN-treated *C. oleifera* pollen tubes and controls. Total RNA was isolated from pistil and pollen tube samples and then reverse-transcribed to synthesize cDNA. The primer of the selected genes was designed using Primer3, with TIF3H1 as the reference gene [63] (Table S2). qRT-PCR was performed on the CFX96 system (Bio-Rad, Hercules, California, American). The relative expression level was calculated using the 2^−ΔΔCt^ method. Each experimental sample was composed of three biological replicates.

### 4.9. Statistical Analysis

The germination and tube length of the pollen were determined using ImageJ software (1.53). Statistical analysis was conducted by employing a one-way ANOVA and *t*-test embedded in SPSS 26. Results were presented as the average values derived from a minimum of three biological replicates and their standard errors. Graphics were presented using Adobe Photoshop 2023 and GraphPad Prism 8.

## 5. Conclusions

The exogenous application of 1000 μmol·L^−1^ MeJA encouraged the self-pollen sprouting and tube elongation of *C. oleifera*, as well as significantly enhanced ovule penetration and fertilization, thereby increasing setting fruit and effectively alleviating the SI of *C. oleifera*. According to transcriptome sequencing analysis, the application of MeJA positively regulated the expression of most genes related to lignin biosynthesis and promoted the deposition of lignin, which increased the germination of pollen. Moreover, transcription factors such as *bHLH* may have played important roles in lignin synthesis, which affected self-pollen tube growth. This research not only provided novel perspectives on the wide application of MeJA but also established a solid groundwork for SI research at the molecular level.

## Figures and Tables

**Table 1:** Effects of methyl jasmonate (MeJA) on the pollen tube growth of *C. oleifera* in vitro.

Concentration	Pollen Germination Rate/%	Length of Pollen Tube/μm	
		2 h	48 h
0 μmol·L^−1^	54.78 ± 1.46 b	789.22 ± 37.73 c	2664.71 ± 22.39 a
0.5 μmol·L^−1^	59.17 ± 1.94 ab	958.06 ± 48.87 b	2698.69 ± 34.71 a
5 μmol·L^−1^	62.66 ± 2.09 a	1010.96 ± 26.54 ab	2732.54 ± 39.42 a
50 μmol·L^−1^	65.14 ± 2.57 a	1126.67 ± 48.97 a	1306.51 ± 66.72 b

Note: The values in the table are mean ± standard error (SE), and different letters represent significant differences by one-way ANOVA with Tukey’s post hoc test (*p* < 0.05).

## Data Availability

The data supporting the findings of this study are available within this article and its Supplementary Materials. The RNA-seq raw data were submitted to the China National Center for Bioinformation (CNCB) (https://bigd.big.ac.cn/gsa/browse/CRA018393 (accessed on 13 September 2024)).

## Supplementary Materials

The following supporting information can be downloaded at https://www.mdpi.com/article/10.3390/ijms251910720/s1.

## References

[1] Zhuang R. (2008). Oil-Tea Camellia in China.

[2] Lin C.-Y., Chen S.-Y., Lee W.-T., Yen G.-C. (2022). Immunomodulatory effect of camellia oil (*Camellia oleifera* Abel.) on CD19 + B cells enrichment and IL-10 production in BALB/c mice. J. Funct. Foods.

[3] Wang T., Wu H.L., Long W.J., Hu Y., Li C., Chen A.Q., Yu R.Q. (2019). Rapid identification and quantification of cheaper vegetable oil adulteration in camellia oil by using excitation-emission matrix fluorescence spectroscopy combined with chemometrics. Food Chem..

[4] Gao J., Ma L., Yin J., Liu G., Ma J., Xia S., Gong S., Han Q., Li T., Chen Y. (2022). Camellia (*Camellia oleifera* bel.) seed oil reprograms gut microbiota and alleviates lipid accumulation in high fat-fed mice through the mTOR pathway. Food Funct..

[5] Wang D., Huo R., Cui C., Gao Q., Zong J., Wang Y., Sun Y., Hou R. (2019). Anticancer activity and mechanism of total saponins from the residual seed cake of *Camellia oleifera* Abel. in hepatoma-22 tumor-bearing mice. Food Funct..

[6] Zhang F., Zhu F., Chen B., Su E., Chen Y., Cao F. (2022). Composition, bioactive substances, extraction technologies and the influences on characteristics of *Camellia oleifera* oil: A review. Food Res. Int..

[7] Quan W., Wang A., Gao C., Li C. (2022). Applications of Chinese *Camellia oleifera* and its By-Products: A Review. Front. Chem..

[8] Yu Z., Wu X., He J. (2022). Study on the antifungal activity and mechanism of tea saponin from *Camellia oleifera* cake. Eur. Food Res. Technol..

[9] Ma B., Huang Y., Nie Z., Qiu X., Su D., Wang G., Yuan J., Xie X., Wu Z. (2019). Facile synthesis of *Camellia oleifera* shell-derived hard carbon as an anode material for lithium-ion batteries. RSC Adv..

[10] Liao T., Yuan D.Y., Zou F., Gao C., Yang Y., Zhang L., Tan X.F. (2014). Self-sterility in *Camellia oleifera* may be due to the prezygotic late-acting self-incompatibility. PLoS ONE.

[11] Gao C., Yuan D., Yang Y., Wang B., Liu D., Zou F. (2015). Pollen Tube Growth and Double Fertilization in *Camellia oleifera*. J. Am. Soc. Hortic. Sci..

[12] Nettancourt D. (2001). Incompatibility and Incongruity in Wild and Cultivated Plants.

[13] Zhang D., Li Y.Y., Zhao X., Zhang C., Liu D.K., Lan S., Yin W., Liu Z.J. (2023). Molecular insights into self-incompatibility systems: From evolution to breeding. Plant Commun..

[14] Broz A.K., Bedinger P.A. (2021). Pollen-Pistil Interactions as Reproductive Barriers. Annu. Rev. Plant Biol..

[15] Chang Y., Gong W., Xu J., Gong H., Song Q., Xiao S., Yuan D. (2023). Integration of semi-in vivo assays and multi-omics data reveals the effect of galloylated catechins on self-pollen tube inhibition in *Camellia oleifera*. Hortic. Res..

[16] Ye M., Peng Z., Tang D., Yang Z., Li D., Xu Y., Zhang C., Huang S. (2018). Generation of self-compatible diploid potato by knockout of S-RNase. Nat. Plants.

[17] Shiba H., Kimura N., Takayama S., Hinata K., Suzuki A., Isogai A. (2000). Alteration of the self-incompatibility phenotype in Brassica by transformation of the antisense SLG gene. Biosci. Biotechnol. Biochem..

[18] O‘Brien M., Kapfer C., Major G., Laurin M., Bertrand C., Kondo K., Kowyama Y., Matton D.P. (2002). Molecular analysis of the stylar-expressed *Solanum chacoense* small asparagine-rich protein family related to the HT modifier of gametophytic self-incompatibility in Nicotiana. Plant J..

[19] Ma L., Zhang C., Zhang B., Tang F., Li F., Liao Q., Tang D., Peng Z., Jia Y., Gao M. (2021). A nonS-locus F-box gene breaks self-incompatibility in diploid potatoes. Nat. Commun..

[20] Lynn A.M., Sullivan L.L., Galen C. (2023). The cost of self-promotion: Ecological and demographic implications of the mentor effect in natural plant populations. New Phytol..

[21] Montalt R., Prosper L., Carmen Vives M., Navarro L., Ollitrault P., Aleza P. (2022). Breakdown of Self-Incompatibility in Citrus by Temperature Stress, Bud Pollination and Polyploidization. Agriculture.

[22] Zhang S., Gao F., Chen D., Gu Z. (2003). The effects of plant growth regulating substances on pollen germination and tube growth in Fengshui pear (*Pyrus serotina*). Acta Bot. Boreali-Occident. Sin..

[23] Zhang X., Liang J., Jing S., Chen F. (2014). Influences of Plant Growth Regulators on Pollen Tube Growth and Germination in “Qinguan” Apple. Chin. Agric. Sci. Bull..

[24] Chang Y., Xu J., Guo X., Yang G., Deng S., Chen Q., Gong H., Song Q., Gong W., Yuan D. (2024). Tannase increases fruit set by interfering with self-incompatibility of *Camellia oleifera*. Ind. Crops Prod..

[25] Yildiz K., Yilmaz H. (2002). Effect of jasmonic acid, ACC and ethephon on pollen germination in strawberry. Plant Growth Regul..

[26] Wu J., Qin Y., Zhao J. (2008). Pollen tube growth is affected by exogenous hormones and correlated with hormone changes in styles in *Torenia fournieri* L.. Plant Growth Regul..

[27] Wu J.-Z., Lin Y., Zhang X.-L., Pang D.-W., Zhao J. (2008). IAA stimulates pollen tube growth and mediates the modification of its wall composition and structure in *Torenia fournieri*. J. Exp. Bot..

[28] Xu J., Chang Y., Gong H., Gong W., Yuan D. (2023). Effects of different exogenous substances on pollen germination and pollen tube growthof *Camellia oleifera*. Acta Agric. Zhejiangensis.

[29] Cheong J.J., Choi Y.D. (2003). Methyl jasmonate as a vital substance in plants. Trends Genet..

[30] Ho T.T., Murthy H.N., Park S.Y. (2020). Methyl Jasmonate Induced Oxidative Stress and Accumulation of Secondary Metabolites in Plant Cell and Organ Cultures. Int. J. Mol. Sci..

[31] Chang L., Wu S., Tian L. (2021). Methyl jasmonate elicits distinctive hydrolyzable tannin, flavonoid, and phyto-oxylipin responses in pomegranate (*Punica granatum* L.) leaves. Planta.

[32] Shi J., Ma C., Qi D., Lv H., Yang T., Peng Q., Chen Z., Lin Z. (2015). Transcriptional responses and flavor volatiles biosynthesis in methyl jasmonate-treated tea leaves. BMC Plant Biol..

[33] Song Q., Gong W., Yu X., Ji K., Jiang Y., Chang Y., Yuan D. (2023). Transcriptome and Anatomical Comparisons Reveal the Effects of Methyl Jasmonate on the Seed Development of *Camellia oleifera*. J. Agric. Food Chem..

[34] Kaushik S., Ranjan A., Singh A.K., Sirhindi G. (2023). Methyl jasmonate reduces cadmium toxicity by enhancing phenol and flavonoid metabolism and activating the antioxidant defense system in pigeon pea (*Cajanus cajan*). Chemosphere.

[35] Muradoğlu F., Yıldız K., Balta F. (2010). Methyl Jasmonate Influences of Pollen Germination and Pollen Tube Growth of Apricot (*Prunus armeniaca* L.). Yznc Yil Niversitesi Tarim Bilim. Derg..

[36] Çetinbaş-Genç A., Vardar F. (2020). Effect of methyl jasmonate on in-vitro pollen germination and tube elongation of *Pinus nigra*. Protoplasma.

[37] Liu Y., Zhou J., Lu M., Yang J., Tan X. (2022). The Core Jasmonic Acid-Signalling Module CoCOI1/CoJAZ1/CoMYC2 Are Involved in Jas Mediated Growth of the Pollen Tube in *Camellia oleifera*. Curr. Issues Mol. Biol..

[38] Zhang Y., Ren Y., Yang D., Liu H., Zhang Y., Wang X., Bai F., Cheng S. (2023). Foliar methyl jasmonate (MeJA) application increased 2-acetyl-1-Pyrroline (2-AP) content and modulated antioxidant attributes and yield formation in fragrant rice. J. Plant Physiol..

[39] Feng K., Yan Y.J., Sun N., Yang Z.Y., Zhao S.P., Wu P., Li L.J. (2024). Exogenous methyl jasmonate treatment induced the transcriptional responses and accumulation of volatile terpenoids in *Oenanthe javanica* (Blume) DC. Int. J. Biol. Macromol..

[40] Song Q., Ji K., Yu X., Chen L., Wang L., Gong W., Yuan D. (2022). Dynamic metabolic and transcriptomic profiling reveal synthetic characters and regulators of flavonoid biosynthesis in *Camellia oleifera* seeds. Ind. Crops Prod..

[41] Wei X., Guan W., Yang Y., Shao Y., Mao L. (2021). Methyl jasmonate promotes wound healing by activation of phenylpropanoid metabolism in harvested kiwifruit. Postharvest Biol. Technol..

[42] Liu C.F., Yang N., Teng R.M., Li J.W., Chen Y., Hu Z.H., Li T., Zhuang J. (2023). Exogenous methyl jasmonate and cytokinin antagonistically regulate lignin biosynthesis by mediating CsHCT expression in *Camellia sinensis*. Protoplasma.

[43] Ji N., Wang J., Li Y., Li M., Jin P., Zheng Y. (2021). Involvement of PpWRKY70 in the methyl jasmonate primed disease resistance against *Rhizopus stolonifer* of peaches via activating phenylpropanoid pathway. Postharvest Biol. Technol..

[44] Liu Q., Luo L., Zheng L. (2018). Lignins: Biosynthesis and Biological Functions in Plants. Int. J. Mol. Sci..

[45] Barakate A., Stephens J., Goldie A., Hunter W.N., Marshall D., Hancock R.D., Lapierre C., Morreel K., Boerjan W., Halpin C. (2011). Syringyl lignin is unaltered by severe sinapyl alcohol dehydrogenase suppression in tobacco. Plant Cell.

[46] Kitin P., Voelker S.L., Meinzer F.C., Beeckman H., Strauss S.H., Lachenbruch B. (2010). Tyloses and phenolic deposits in xylem vessels impede water transport in low-lignin transgenic poplars: A study by cryo-fluorescence microscopy. Plant Physiol..

[47] Eudes A., Pollet B., Sibout R., Do C.T., Séguin A., Lapierre C., Jouanin L. (2006). Evidence for a role of AtCAD 1 in lignification of elongating stems of *Arabidopsis thaliana*. Planta.

[48] Vanholme R., Demedts B., Morreel K., Ralph J., Boerjan W. (2010). Lignin Biosynthesis and Structure. Plant Physiol..

[49] Zhong T.X., Tang R., Song J.L., Fu C.C., Liu Y., Zhou C.C., Zhang X.Q., Chen S., Xie X.M. (2018). Vascular preferential activity of the *Pennisetum purpureum* cinnamyl alcohol dehydrogenase promoter in transgenic tobacco plants. Plant Physiol. Biochem..

[50] Kim J.I., Hidalgo-Shrestha C., Bonawitz N.D., Franke R.B., Chapple C. (2021). Spatio-temporal control of phenylpropanoid biosynthesis by inducible complementation of a cinnamate 4-hydroxylase mutant. J. Exp. Bot..

[51] El Houari I., Van Beirs C., Arents H.E., Han H., Chanoca A., Opdenacker D., Pollier J., Storme V., Steenackers W., Quareshy M. (2021). Seedling developmental defects upon blocking CINNAMATE-4-HYDROXYLASE are caused by perturbations in auxin transport. New Phytol..

[52] Yuan M., Shu G., Zhou J., He P., Xiang L., Yang C., Chen M., Liao Z., Zhang F. (2023). AabHLH113 integrates jasmonic acid and abscisic acid signaling to positively regulate artemisinin biosynthesis in *Artemisia annua*. New Phytol..

[53] Bernardini A., Lorenzo M., Chaves-Sanjuan A., Swuec P., Pigni M., Saad D., Konarev P.V., Graewert M.A., Valentini E., Svergun D.I. (2021). The USR domain of USF1 mediates NF-Y interactions and cooperative DNA binding. Int. J. Biol. Macromol..

[54] Liu Y., Xi W., Wang X., Li H., Liu H., Li T., Hou J., Liu X., Hao C., Zhang X. (2023). TabHLH95-TaNF-YB1 module promotes grain starch synthesis in bread wheat. J. Genet. Genom..

[55] Hu G., Gao C., Fan X., Gong W., Yuan D. (2020). Pollination Compatibility and Xenia in *Camellia oleifera*. HortScience.

[56] Gao C., Yuan D., Yang Y., Wang B., Liu D., Zou F., Tan X. (2015). Anatomical Characteristics of Self-Incompatibility in *Camellia oleifera*. Sci. Silvae Sin..

[57] Gao C., Yuan D.Y., Wang B.F., Yang Y., Liu D.M., Han Z.Q. (2015). A cytological study of anther and pollen development in *Camellia oleifera*. Genet. Mol. Res..

[58] Foster C.E., Martin T.M., Pauly M. (2010). Comprehensive compositional analysis of plant cell walls (*Lignocellulosic biomass*) part I: Lignin. J. Vis. Exp. JoVE.

[59] Lin P., Wang K., Wang Y., Hu Z., Yan C., Huang H., Ma X., Cao Y., Long W., Liu W. (2022). The genome of oil-Camellia and population genomics analysis provide insights into seed oil domestication. Genome Biol..

[60] Szklarczyk D., Kirsch R., Koutrouli M., Nastou K., Mehryary F., Hachilif R., Annika G.L., Fang T., Doncheva N.T., Pyysalo S. (2023). The STRING database in 2023: Protein–protein association networks and functional enrichment analyses for any sequenced genome of interest. Nucleic Acids Res..

[61] Liao F., Wang L., Yang L.-B., Zhang L., Peng X., Sun M.-X. (2013). Antisense Oligodeoxynucleotide Inhibition as an Alternative and Convenient Method for Gene Function Analysis in Pollen Tubes. PLoS ONE.

[62] Mizuta Y., Higashiyama T. (2014). Antisense gene inhibition by phosphorothioate antisense oligonucleotide in Arabidopsis pollen tubes. Plant J..

[63] Chang Y., Hu S., Xu J., Gong H., Guo X., Song Q., Gong W., Yuan D. (2023). Identification of reference genes provides insights into the determinants of self-incompatibility in *Camellia oleifera*. Sci. Hortic..

