# Supplementary material for: Methyl Jasmonate (MeJA) Promotes the Self-Pollen Tube Growth of Camellia oleifera by Regulating Lignin Biosynthesis
Source: Int J Mol Sci. 2024 Oct 5;25(19):10720. doi: 10.3390/ijms251910720 (PMC11476367; doi:10.3390/ijms251910720)
Supplement: Supplementary file 1 [file ijms-25-10720-s001.zip › Supplementary Files.pdf]

Methyl Jasmonate (MeJA) Promotes the Self-Pollen Tube Growth of *Camellia oleifera* by Regulating Lignin Biosynthesis

Yihong Chang †, Xinmiao Guo †, Honggang Xu, Qixiao Wu, Anqi Xie, Zhixuan Zhao, Ruijie Tian, Wenfang Gong * and Deyi Yuan *

Supplementary Files

Figures S1 and S2

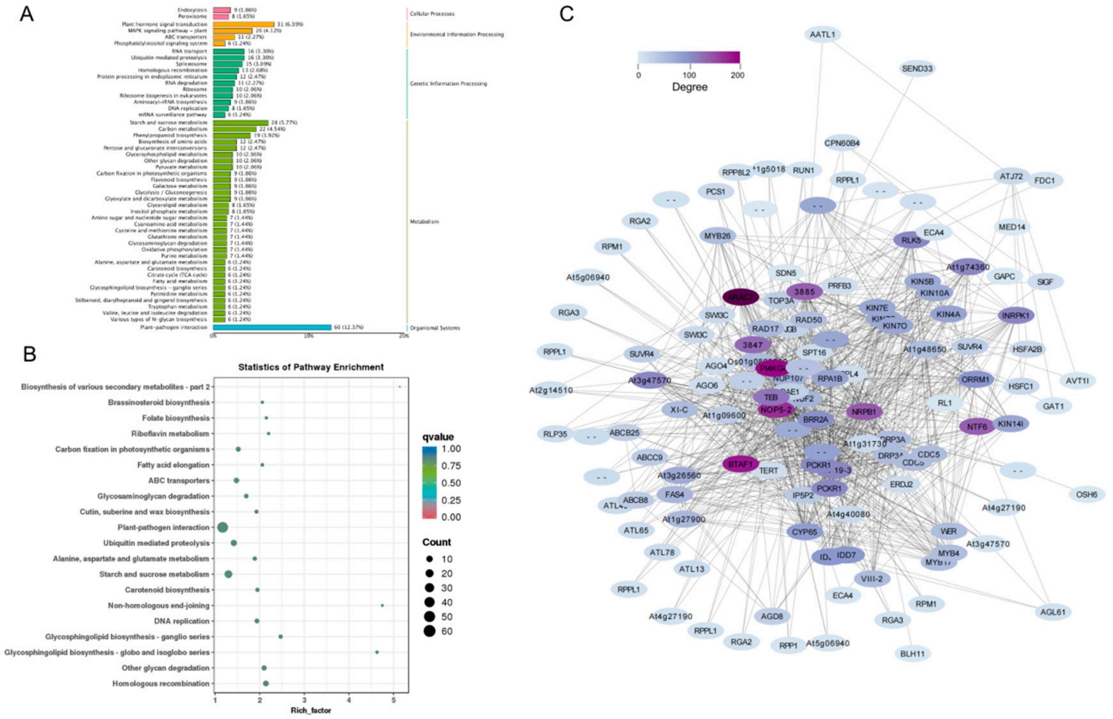

Figure S1 Functional analysis of 2015 CK group-specific genes. KEGG function categorization (A) and enrichment (B) diagram; (C) Gene interaction network diagram. Dark colors represent hub genes.

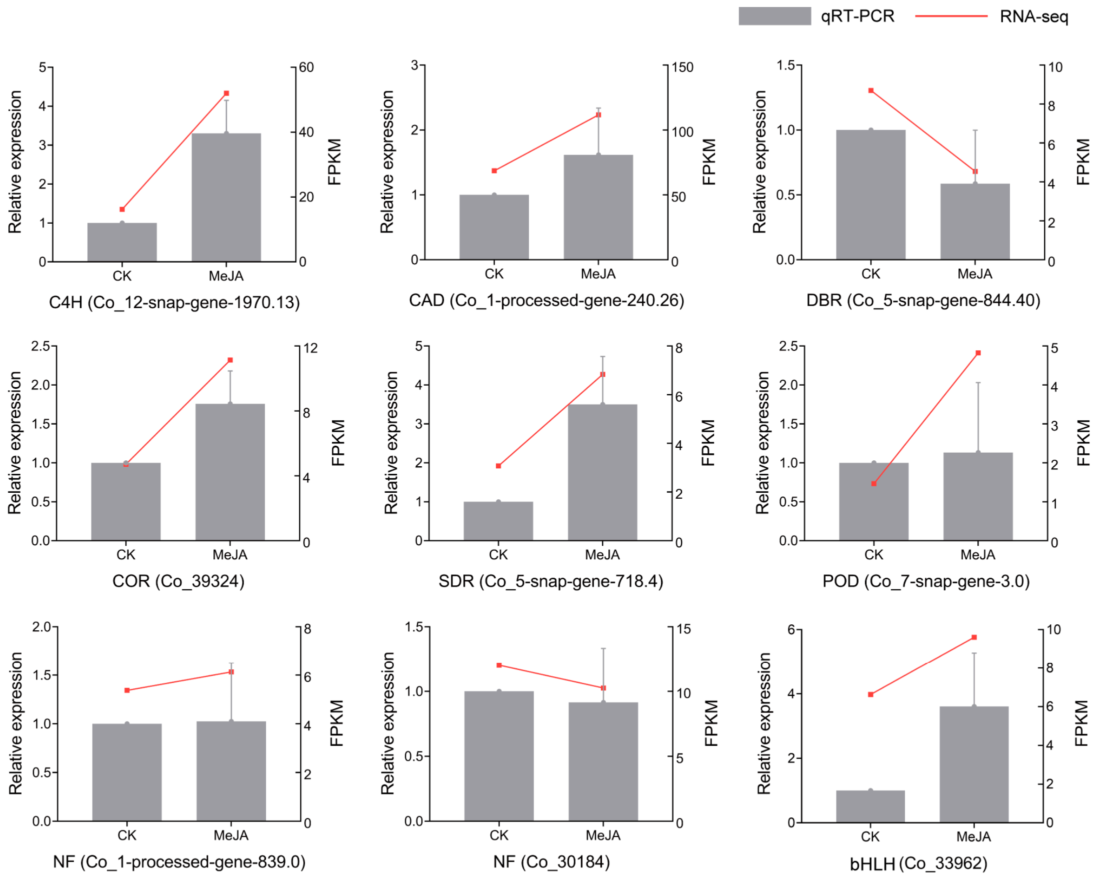

Figure S2 qRT-PCR validation of six DEGs and three TFs. The error bars display the standard error of the mean (n = 3).
